# A Combinational Strategy Mitigated Old-Aged Petroleum Contaminants: Ineffectiveness of Biostimulation as a Bioremediation Technique

**DOI:** 10.3389/fmicb.2021.642215

**Published:** 2021-02-25

**Authors:** Hamidreza Garousin, Ahmad Ali Pourbabaee, Hossein Ali Alikhani, Najmeh Yazdanfar

**Affiliations:** ^1^Biology and Biotechnology Lab, Department of Soil Science, University College of Agriculture and Natural Resources, University of Tehran, Karaj, Iran; ^2^Iranian Institute of R&D in Chemical Industries (IRDCI) (ACECR), Tehran, Iran

**Keywords:** total petroleum hydrocarbons, aged-contaminated soil, bioremediation, soil biological factors, correlation coefficient

## Abstract

Hydrocarbon contamination emerging from the crude oil industrial-related activities has led to severe environmental issues. Prolonged contamination with the constant infiltration of crude oil into the soil is a severe problem in remediating contaminated soils. Hence, the current study focuses on comparing various bioremediation strategies, thereby isolating native bacteria competent to reduce TPH in both liquid and microcosm environments in an old-aged petroleum hydrocarbon contaminated soil. Assays in the modified 6SW-Vit medium after 7 days of incubation revealed that *Bacillus altitudinis* strain HRG-1 was highly hydrophobic and had a suitable ability to decrease surface tension (40.98%) and TPH (73.3%). The results of biodegradation in the microcosm proved that among the designated treatments, including bio-stimulated microcosm (SM), bacterialized microcosm (BM), a combined bio-stimulated microcosm and bacterialized microcosm (SB), and natural attenuation (NA), the SB treatment was the most effective in mitigating TPH (38.2%). However, the SM treatment indicated the lowest TPH biodegradation (18%). Pearson correlation coefficient among microcosm biological indicators under investigation revealed that soil basal respiration had the highest correlation with the amount of residual TPH (*r* = −0.73915, *P* < 0.0001), followed by the microbial population (*r* = −0.65218, *P* < 0.0001), catalase activity (*r* = 0.48323, *P* = 0.0028), polyphenol oxidase activity (*r* = −0.43842, *P* = 0.0075), and dehydrogenase activity (*r* = −0.34990, *P* = 0.0364), respectively. Nevertheless, considering the capability of strain HRG-1 and the higher efficiency of the combined technique, their use is recommended to diminish the concentration of petroleum hydrocarbons in hot and dry contaminated areas.

## Introduction

Total petroleum hydrocarbons are one of the most momentous contaminants in the environment. Considering the harmful effects of crude oil leakage on the region’s ecosystem, it is necessary to remove them from the environment. One of the most effective, low-cost, and applicable techniques to remove contaminants is called bioremediation. Bioremediation denotes the use of living indigenous or exogenous microorganisms with the property of degradation of contaminants or improvement of various physical-chemical soil conditions to stimulate the growth of efficient microorganisms (Wilson and Jones, 1993; MarGesin et al., 2000). Solving the contamination problem has attracted many researchers’ attention in recent years; however, the aging of organic contaminants and the successive entry of petroleum hydrocarbons into the soil have been less discussed and investigated.

Numerous studies have reported using different bioremediation strategies in petroleum-contaminated soils, each of which presenting different results. For instance, Liu et al. (2011) said bioaugmentation as a superior strategy, while in another study, Wu et al. (2019) considered biostimulation a preferable approach. In other researchers’ works, the combined bioaugmentation and biostimulation has been proposed as the most efficient strategy (Varjani and Upasani, 2019).

The core problem facing bioremediation is the aging phenomenon of organic contaminants in the soil. As time passes, it becomes laborious to separate the hydrocarbon contaminant from the soil matrix. With these contaminants’ infiltration into soil organic matter and soil pores, their extraction ability and accessibility for microorganisms and other environmental receptors are reduced (Tang et al., 2012). Due to the complexity of old-contaminants’ behavior in the soil, more robust strategies are needed for the bioremediation of these contaminants. Many studies have been conducted on artificial soil contamination (Kang et al., 2010; Xu et al., 2018; Baoune et al., 2019), and soils with extremely old and natural contamination have not been investigated enough, and if so, the duration of the contamination has not been clearly mentioned (Dandie et al., 2010; Dong et al., 2013; Lominchar et al., 2018; Liu et al., 2019; Gou et al., 2020; Wang et al., 2020). However, there are many areas suffering from long-term contamination of crude oil and its products, and failure to address this issue can exert irreversible effects. Therefore, the simultaneous use of different bioremediation strategies can be more effective in solving this problem.

Another problem of contaminated soils in hot-dry areas is the salinity effect. High salinity inhibits the growth of microorganisms, increases the adaptation time of microorganisms to new conditions, and reduces the intensity and amount of biodegradation (Qin et al., 2012). Nonetheless, to analyze soil contaminants’ changes resulting from the use of single or combined bioremediation techniques, various biological indicators such as soil basal respiration, microbial population, and enzymatic activity have been examined (Abed et al., 2015; Wu et al., 2017). Numerous studies have reported a strong correlation (positive or negative) between the amount of petroleum hydrocarbons and soil enzymes (Ma et al., 2014; Khan et al., 2015; Ebadi et al., 2017). However, the quantity of the correlation coefficient between soil enzymes and the amount of hydrocarbon contaminants, especially in old-aged contaminated soils, has been less discussed. To evaluate the result of soil bioremediation, it is necessary to consider the amount of residual hydrocarbons, and to determine the effect of contaminants on the biological activity of soil microorganisms. This elaborates an insight into the health condition of soil microorganisms (MarGesin et al., 2000).

The present study aimed to reduce TPH in both microcosm and culture media by a capable bacterial strain and add various supplements to increase biodegradation of contaminants in an old-aged petroleum contaminated saline-sodic soil (more than 60 years of contamination). Additionally, various soil biological indicators such as several enzyme activities, soil basal respiration, and microbial population were investigated to achieve the most appropriate one in showing TPH variations in an old-aged petroleum contaminated soil. To that end, the relationship between the soil biological characteristics and the amount of residual TPH was examined using the Pearson product-moment correlation.

This study was conducted to answer fundamental questions, including (a) which bioremediation strategy has a more influential role in reducing TPH in a highly contaminated aged soil? (b) could this soil with such a long history of contamination be remediated by only one strain or not?

## Materials and Methods

### Soil Sampling and Characterization

Sampling was carried out in crude oil-contaminated saline and sodic soil with more than 60 years of contamination (ranging from 0 to 20 cm depth) around one of the oil exploitation wells located in Ahvaz oilfield (southwestern Iran). The samples were transferred to the laboratory and stored at 4°C until the experiments were performed. The main physical-chemical parameters of the soil were investigated using standard methods (Sparks, 1996). Table 1 presents the properties of the contaminated soil.

**TABLE 1:** Physicochemical and biological petroleum-contaminated soil characteristics.

Characteristics	Unit	Amount
Sand	**%**	66 ± 3.26
Silt	**%**	24 ± 2.94
Clay	**%**	10 ± 3.74
Electrical conductivity (EC)	dS/m	80.6 ± 0.94
pH		7.52 ± 0.08
Field capacity (FC)	**%**	26.22 ± 1.59
Sodium adsorption ratio (SAR)	(mmol/l)^1/2^	258.92 ± 1.63
Total nitrogen	**%**	0.261 ± 0.02
Organic carbon	**%**	8.775 ± 0.33
Total phosphorus	**%**	0.0247 ± 0.001
C/N/P		356/10/1
Na^+^	mg/kg	32660 ± 254.32
K^+^	mg/kg	19.154 ± 1.25
Ca^2+^	mg/kg	1200 ± 48.98
Mg^2+^	mg/kg	729 ± 29.76
Fe^2+^	mg/kg	7.43 ± 0.17
Mn^2+^	mg/kg	19.79 ± 0.59
Cu^2+^	mg/kg	0.67 ± 0.05
Zn^2+^	mg/kg	0.68 ± 0.03
B	mg/kg	1.354 ± 0.03
SO_4_^2–^	meq/l	56.02 ± 6.89
Cl^–^	meq/l	817 ± 25.93
CO_3_^2–^	meq/l	
HCO_3_^–^	meq/l	6.4 ± 0.82
Bacterial population	CFU/g	1.4 ± 0.368×10^4^
TPH	mg/kg	82895 ± 5330.13

*Data are mean of three replicates (±SD).*

### Culture Medium and Materials

The modified 6SW-Vit medium (McGenity and Gramain, 2010) containing three solutions was used for biodegradation assays. Solution A contained (*g* 800 ml^–1^) 30 NaCl, 10 MgCl_2_.6H_2_O, 2.53 KCl, 2.18 Na_2_SO_4_, 20 ml Tris–HCl (1 M; pH 7.6), 0.5 yeast extract. Solution B contained (ml 100 ml^–1^) 0.6 H_3_BO_3_ (400 mM), 0.7 SrCl_2_ (400 mM), 0.7 NaF (70 mM), 1 NH_4_Cl (500 mM), 1 KH_2_PO_4_ (100 mM), two trace element solution SL-10, and solution C contained (ml 100 ml^–1^) 10 CaCl_2_.2H_2_O (1 M). First, the solutions were separately autoclaved and thereafter mixed, when they were cooled down. Trace element solution SL-10 contained (per L) 10 ml HCl (25%; 7.7 M), 1.5 g FeCl_2_.4H_2_O, 70 mg ZnCl_2_, 100 mg MnCl_2_.4H_2_O, 6 mg H_3_BO_3_, 190 mg CoCl_2_.6H_2_O, 2 mg CuCl_2_.2H_2_O, 24 mg NiCl_2_.6H_2_O, 36 mg Na_2_MoO_4_.2H_2_O. Finally, pH was adjusted to the range of 7–7.2. All solvents, acids, inorganic salts and Tween 80 nonionic surfactant were purchased from Merck Co., and the heavy Persian crude oil was used in this study.

### Enrichment and Isolation Process

During the enrichment process, 10 g of crude oil-contaminated soil was transferred to a 500-ml flask containing 100 ml of the modified 6SW-Vit medium with 1% crude oil as a sole source of carbon. The samples were incubated for 7 days in a shaker incubator (150 rpm and 40°C). To complete the enrichment process, 10 ml of the enriched medium was transferred to 90 ml of the fresh saline medium under all previous conditions, and this operation was repeated four times. Upon completion of the enrichment process, a serial dilution was prepared from the final enriched culture medium and transferred to plates containing the modified 6SW-Vit agar culture medium. To select the superior isolate, the ability to degrade crude oil was assessed by pure isolates isolated from the enrichment process using the turbidity method (spectrophotometer JENWAY 6705 UV/Vis) in the 6SW-Vit culture medium (Kumar et al., 2008).

### Biochemical and 16S rRNA Characterization

For primary identification and classification of the bacterial strain, various biochemical analyzes were conducted (Krieg and Manual, 1984). Qiagen kit Cat. No. 51504 was used to extract the total genomic DNA of the bacterial strain. The 16S rRNA gene of pure isolates was amplified, and then the purified PCR products were sequenced by Microsynth Company (Balgach, Switzerland). EzBioCloud and BLAST databases were used to determine the similarity of the sequence to other sequences of known bacteria.

### Analyses in Culture Medium

#### Evaluation of Crude Oil Biodegradation

To select the highest biodegraded concentration of crude oil, four crude oil concentrations [0.25, 0.5, 1, and 2 (V / V)] were designated, and according to the spectrophotometric method (Rahman et al., 2002), the optimal concentration was determined. To more accurately investigate biodegradation, the selected optimal concentration of crude oil was poured in a 250-ml Erlenmeyer flask containing 50 ml of a modified 6SW-Vit medium, and the superior isolate (4% V) was inoculated in it with the initial population of 4.1 × 10^7^ CFU/ml. The specimens were incubated in a shaker incubator (150 rpm and 40°C) for 7 days. After the incubation period, to evaluate the reduction in TPH, the specimens were dehydrated and deasphaltenated by anhydrous sodium sulfate and a PTFE syringe filter (0.45 μm, membrane solution), respectively. A GC-FID (Gas chromatography flame ionization detector) device (AGILENT, 6890N, HP5 column, United States) was used to measure the residual concentration of TPH.

#### Cell Surface Hydrophobicity Assays

Bacterial adhesion to hydrocarbons (BATH) test was used to measure cells’ hydrophobicity with two different types of solvents (hexane as a nonpolar and dichloromethane as a polar solvent). The trend of changes in the hydrophobicity of the cell surface was examined every 24 h over a week. Bacterial cells were precipitated by centrifuging (TOMY SEIKO CO., LTD, RS-20IV) at 6,000 g for 20 min and washed twice with saline solution. The cells were kept in a buffer saline solution (pH = 7) containing 16.9 g L^–1^ K_2_HPO_4_, 7.3 g L^–1^ KH_2_PO_4_, 1.8 g L^–1^ urea, and 0.2 g L^–1^ MgSO_4_.7H_2_O to achieve OD = 0.3 at 600 nm wavelength. Furthermore, 3 ml of the prepared culture medium and the same amount of hydrocarbons (n-hexane and dichloromethane) were poured into the test tubes, and after 5 min, they were mixed with a vortex at high speed for 60 s. After keeping the test tubes at room temperature (15 min) and measuring their turbidity at 600 nm, the percentage of hydrophobicity was calculated using the following equation (Rosenberg et al., 1980).

$$\mathrm{Percentage}\mathrm{of}\mathrm{hydrophobicity}=\left(1-\left(\text{X}/X_{0}\right)\right)\times 100$$

*X* is the optical density of the aqueous phase after addition of n-hexane and dichloromethane. *X*_0_ is the optical density of the primary aqueous phase.

The percentage of the cells moved to the hydrocarbon phase indicates cell-surface hydrophobicity.

#### Biosurfactant Production Analyses and Surface Tension Measurement

The oil displacement analysis was conducted by first pouring 40 ml of distilled water into a petri dish (150 mm diameter) and then pouring 40 μl of crude oil onto it to form a thin layer of oil on the surface of the distilled water. Additionally, 10 μl of the cell-free modified 6SW-Vit medium were gently poured from a suitable height onto a Petri dish’s center. At the end, the diameter of the clear halo zone was measured as a qualitative indicator of biosurfactant production (Morikawa et al., 2000).

To investigate the hemolytic activity of the cells, the blood agar medium (5% sheep’s blood) was used to culture the bacterial strain. Then, it was incubated at 30°C for 48 h. The clear zone formed around the colony represents the production of biosurfactant (Carrillo et al., 1996).

The Wilhelmy plate method (KRUSS tesiometer) was also used to determine surface tension according to (Rosenberg et al., 1980).

### Analyses in Microcosm

#### Microcosm Design

Four treatments were designed in this study including 1: bio-stimulated microcosm (SM) with substances such as Tween 80 surfactant (1% V/W), NH_4_NO_3_ as a source of nitrogen and KH_2_PO_4_ as a source of phosphorus, 2: bacterialized microcosm (BM) (10% V/W) with an initial population of 5.4 × 10^8^ CFU/ml, 3: combined bio-stimulated microcosm and bacterialized microcosm (SB) and 4: natural attenuation (NA). To prepare the designed treatments, 100 g of the contaminated soil was poured into each jar, and the humidity in all treatments was considered 65% field capacity (FC). The C/N/P ratio of the soil was adjusted to 100/10/2 in the bio-SM. All treatments were incubated in a dark environment at 40°C for 60 days, and every 20 days, the activity of soil enzymes (dehydrogenase, polyphenol oxidase, and catalase), soil basal respiration, soil bacterial population, and biodegradation rate of TPH were examined. The treatments were exposed to the open air for 10 min every 7 days to provide oxygen, and the moisture content was adjusted to the desired amount every 7 days.

#### Enzymatic Analyses

To determine dehydrogenase activity, 3 g of the moist soil sample was thoroughly mixed with 30 mg CaCO_3_, and then 0.5 ml of 3% triphenyl tetrazolium chloride (TTC) and 1.25 ml distilled water were added to each sample and incubated for 24 h at 37°C. Afterward, 10 ml of methanol was added to each sample and thoroughly mixed by Vortex, and then centrifuged at 4,000 rpm (15 min) for the precipitation process. Absorbance in the extract was measured at 485 nm (Casida et al., 1964). Eventually, dehydrogenase activity was determined as μg TPF g^–1^ moist soil h^–1^.

To evaluate polyphenol oxidase activity, 1 g of the moist soil was poured into 50-ml glass falcons. Then 10 ml of the pyrogalic acid solution (1%) was added to it, mixed well and incubated at 30°C for 1 h. Subsequently, 2.5 ml of HCl (0.5 M) was gently added to it, and the resulting solution was extracted by ether with a separatory funnel. The organic phase was collected and spectrophotometrically read at 430 nm. The control had all the above conditions, and only 10 ml of distilled water was used instead of 10 ml of pyrogalic acid (Ma et al., 2003). Polyphenol oxidase activity was expressed as mg purpurogallin g^–1^ moist soil h^–1^.

To assay catalase activity, 2 g of the moist soil with 40 ml of distilled water, and 5 ml of H_2_O_2_ (0.3%) were mixed and shaken (150 rpm, 20 min) and immediately after shaking, 5 ml of H_2_SO_4_ (3 M) was added to prevent further decomposition of H_2_O_2_. Finally, the resulting product was filtered, and 25 ml of the filtered solution was titrated with KMnO_4_ (0.1 N) (Lin et al., 2009). Catalase activity was conveyed as ml KMnO_4_ g ^–1^ moist soil h^–1^.

#### Soil Basal Respiration and Bacterial Population

100 g of the contaminated soil was poured in sterile glass jars closed with lids and then, NaOH (0.2 mol/l) was used to trap the CO_2_ evolved from the soil incubation experiment. Finally, The NaOH solution was titrated by 0.1 M HCl, and it was presented as mg CO_2_/kg soil/h (Schinner et al., 2012).

Soil microbial population was evaluated using the standard plate count (SPC) method with plates containing the modified 6SW-Vit agar culture medium.

#### TPH Biodegradation

Soil treatments were extracted every 20 days by ultrasound (Hielscher, UP400S, 400 W, 24 KHz) to measure biodegradation of TPH. Briefly, 4 g of the contaminated soil was mixed with a mixture of dichloromethane and acetone solvents (10:10 ml). For the dehydration process, 2 g of anhydrous sodium sulfate was added to the samples. The samples were then extracted (20 min, twice) and centrifuged (4,000 rpm, 10 min). The organic phase was transferred to other glass Falcons and spectrophotometrically read at a wavelength of 420 nm (Rahman et al., 2002).

### Statistical Analyses

This study was conducted as a factorial experiment in a completely randomized design (CRD) with three replications. Before analysing variance, normality of distribution of the data (Jarque-Bera test), and homogeneity of variances (Levene’s test) were analyzed using XLSTAT 2019, and SPSS 21, respectively. Two-way analysis of variance (ANOVA) was conducted to evaluate the interaction between microcosm treatments and different times (SAS 9.4). Duncan’s post hoc test was applied to compare among multiple groups (SAS 9.4). Pearson product-moment correlation was conducted to determine the relationships between the studied microcosm biological characteristics and the amount of residual TPH (SAS 9.4). Probability levels of 0.01 were considered statistically significant. Microsoft Excel software 2013 was used to draw graphs.

## Results and Discussion

### Selection and Identification of Bacterial Strain

The enrichment process only yielded two isolates after 5 weeks. The turbidity results indicated that merely one of the two isolates was efficient enough to create turbidity so that it was selected as the superior isolate for further studies. The 16S rRNA gene sequencing results signified our strain’s highest affinity (100%) with *Bacillus altitudinis* strain 41KF2b^(T)^ under the accession number of ASJC01000029. The strain was registered in the gene bank under *B. altitudinis* strain HRG-1^(T)^ under the accession number of MN590432. This strain was seamlessly nurtured in the pH range of 5.5 to 8.5 (optimum pH = 7), NaCl concentrations of 0 to 5% (optimum 3%) and temperature range of 20 to 55°C (optimum 30 °C). Table 2 shows some morphological and biochemical characteristics of the strain HRG-1.

**TABLE 2:** Some morphological and biochemical characteristics of HRG-1 strain.

Test	Result
Shape	Rod-shaped
Color	Creamy
Gram reaction	+
Spore	+
Motility	+
Fermentations	
Glucose	+
Maltose	−
Mannitol	+
Fructose	+
Arabinose	−
Lactose	−
Glycerol	−
Casein hydrolysis	−
Nitrate reduction	−
Citrate utilization	+
Oxidative/Fermentative	O/F
Catalase	+
Oxidase	+
Lipase	+
Urease	−
Lecithinase	−
α-amylase	−

### Biodegradation of TPH in the Modified 6SW-Vit Medium

**FIGURE 1:**
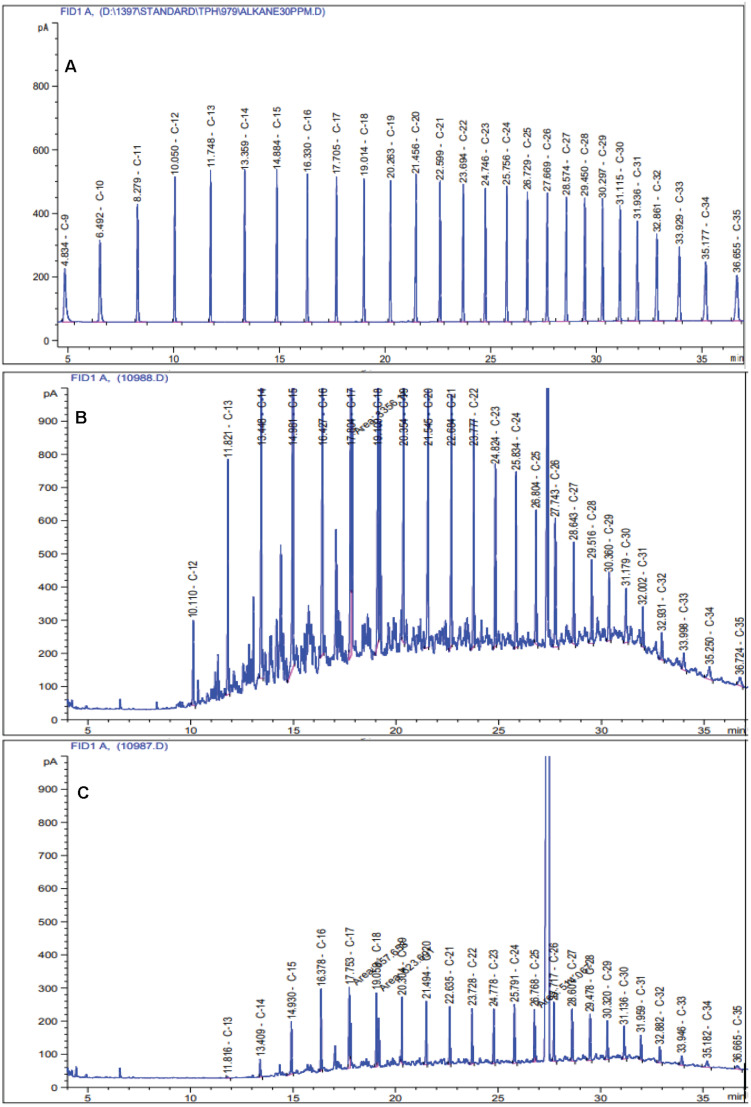
Chromatograms of residual TPH in the modified 6SW-Vit medium after 7 days of incubation at 40 °C temperature. **(A)** standard mix solution of TPH, **(B)** non-biodegraded control, and **(C)** biodegraded treatment.

Among the four selected crude oil concentrations, 0.25% displayed the highest amount of degradation in the liquid medium; hence, it was selected as the optimal concentration (Supplementary Figure 1). The GC-FID results revealed that the HRG-1 strain could degrade 73.3% ± 6.58 of TPH in the modified 6SW-Vit medium after 7 days (Figure 1). In one study conducted under optimal conditions, Liu et al. (2016), reported that after 5 days, *Bacillus licheniformis* strain Y-1 degraded 60.2% of crude oil. In another study, after 12 days of incubation, *Dietzia* sp. Strain CN-3 efficiently degraded 91.6% of crude oil (0.5% w/v) in the liquid medium (180 rpm, 30°C) (Chen et al., 2017).

### Bacterial Adhesion to Hydrocarbons Test

**FIGURE 2:**
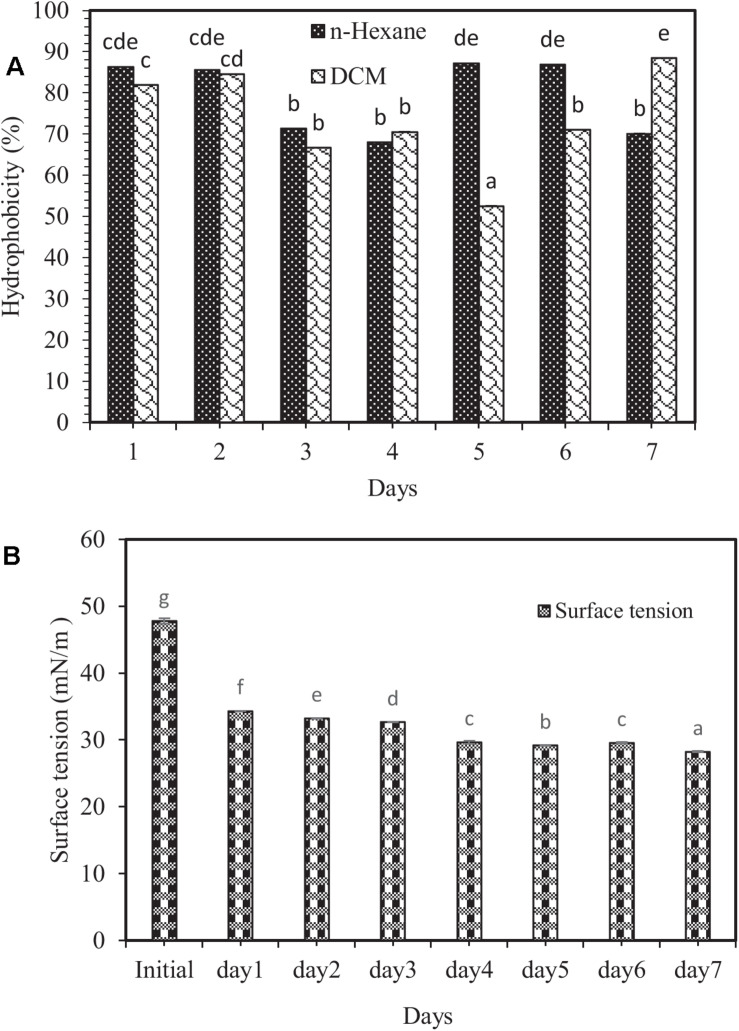
**(A)** Changes in the percentage of cell surface hydrophobicity, and **(B)** surface tension variations in the modified 6SW-Vit medium amended with crude oil at 7 days. Means with the same letters are not significantly different at (*P* ≤ 0.01). Error bars are not clearly visible because they are shorter than the symbol size and represent the standard deviation (*n* = 3). DCM and n-hexane are the abbreviations of dichloromethane and normal-hexane, respectively.

Biosurfactants can be adsorbed by the cell surface, thereby increasing cell surface hydrophobicity (Ahimou et al., 2000). Cell surface hydrophobicity test is one of the exciting experiments in environmental engineering and other microbial disciplines (Saini, 2010). The results showed that the highest and lowest hydrophobicity levels were related to dichloromethane solvent (88.44%) on the 7 day, and (52.55%) on the 5 day of the experiment, respectively (Figure 2A). No significant difference between dichloromethane and n-hexane was observed, except on the 5th, 6th, and 7th days. The reason that such changes in target hydrocarbons result in changes in hydrophobicity is probably the different viscosity of hydrocarbons as well as the size of the droplets formed during the mixing process (Rosenberg, 1984).

### Evaluation of Oil Displacement, Hemolytic Activity and Changes in Surface Tension

The oil displacement test is a qualitative study of the presence of biosurfactants in solution. The diameter of the clear zone on the surface is directly related to the presence of biosurfactants (Ibrahim et al., 2013). Our study indicated that the diameter of the clear zone on the thin layer of oil was 31 mm, suggesting the production of biosurfactants during the biodegradation of crude oil (Supplementary Figure 2a).

Another hint for biosurfactant production is the lysis of red blood cells; HRG-1 strain did it best and caused hemoglobin breakdown (Supplementary Figure 2b). Consistent with the classifications proposed by Carrillo et al. (1996) and creating a white and clear zone around the colony, this strain was placed in the type of β-hemolysis.

One of the core indicators of biosurfactants’ production is a decrease in surface tension (Diniz Rufino et al., 2014). Biosurfactants boost hydrocarbon compounds’ surface area, thereby allowing further degradation (Batista et al., 2006; Hassanshahian et al., 2014). In this investigation, changes in surface tension in the modified 6SW-Vit medium during 7 days of biodegradation exhibited the highest surface tension related to the initial time (47.81 mN/m), while the lowest one to the 7th day (28.22 mN/m). However, after 7 days, the surface tension dropped by 40.98% (Figure 2B). In a study, Goswami and Deka (2019) reported that after 48 h of incubation, biosurfactant producing bacterium (*B. altitudinis* strain MS16) reduced the surface tension of the culture medium from 72.8 to 32.3 mN/m.

### Biological Assays in Crude Oil-Contaminated Microcosm

**FIGURE 3:**
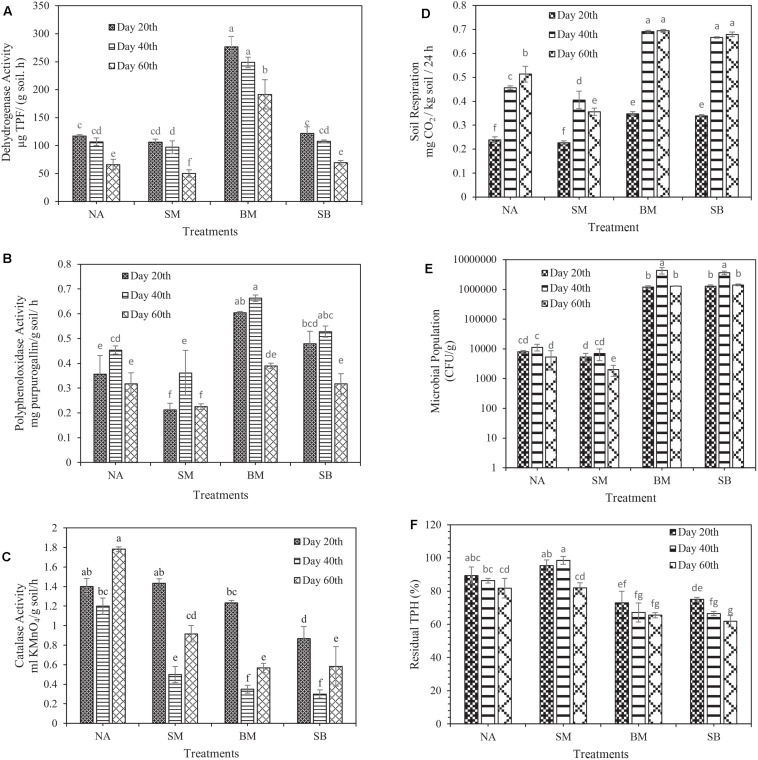
Evaluation of changes in biological characteristics of crude oil-contaminated soil and biodegradation of TPH during 60 days of incubation. **(A)** Dehydrogenase activity, **(B)** polyphenol oxidase activity, **(C)** catalase activity, **(D)** soil basal respiration, **(E)** microbial population, and **(F)** biodegradation of TPH. (NA, natural attenuation; SM, bio-stimulated microcosm; BM, bacterialized microcosm, and SB, combined bio-stimulated microcosm and bacterialized microcosm). Means with the same letters are not significantly different at (*P* ≤ 0.01). Error bars indicate the standard deviation (*n* = 3).

In some cases, when the issue of oxidation of soil organic matter is raised, dehydrogenase activity is a worthy indicator for organic matter changes, particularly in petroleum-contaminated soils, and it is part of the respiratory process of soil microorganisms (Das and Varma, 2010). The results of this study displayed that the amount of dehydrogenase activity (in all treatments) at the end of the first 20 days was higher than that at the beginning of the experiment (99.6 ± 2.82 μg TPF g^–1^ moist soil h^–1^). Nevertheless, in all treatments, the highest and lowest dehydrogenase activity was observed in the first and third 20 days, respectively. Changes in dehydrogenase activity throughout the experiment had a decreasing trend, being consistent with the notions reported by Chen et al. (2019). The highest dehydrogenase activity during the experimental period was related to the BM treatment in the first 20 days (276.5 μg TPF g^–1^ moist soil h^–1^). The lowest was related to the SM treatment in the third 20 days (50.26 μg TPF g^–1^ moist soil h^–1^). The results clearly revealed that the application of biostimulation prevented the increase of dehydrogenase activity during the incubation period (Figure 3A).

Polyphenol oxidase plays a crucial role in the degradation of aromatic hydrocarbons (phenolic compounds); it is an essential indicator of microbial activity, particularly in contaminated soils (Dindar et al., 2015). The results of the present study revealed that the polyphenol oxidase activity in all treatments (except SB) in the first 20 days of incubation was increased compared to its initial time (0.256 ± 0.019 mg purpurogallin g^–1^ moist soil h^–1^). During 60 days of incubation, the lowest and the highest polyphenol oxidase activity was associated with the SM treatment (0.212 mg purpurogallin g^–1^ moist soil h^–1^) in the first 20 days and the BM treatment (0.662 mg purpurogallin g^–1^ moist soil h^–1^) in the second 20 days, respectively (Figure 3C). In their study, Wu et al. (2017) found that polyphenol oxidase activity in all treatments reached its maximum on the 7th day. Then, it rapidly dropped toward the end of the experiment, being in line with the polyphenol oxidase activity changes in our study. It is noteworthy that similar to dehydrogenase activity, the application of biostimulation prohibited the increase of polyphenol oxidase activity during the incubation period.

Catalase diminishes the harmful effects of heavy metals and hydrocarbons by performing its catalytic function in the degradation of H_2_O_2_; thus, it is interesting to study it in contaminated soils (Achuba and Okoh, 2014). Its unique characteristics such as time-consuming performance, low cost and ease of use have made it highly useful to study catalase activity (Chelikani et al., 2004). The results of this study presented that the activity of catalase was decreased from the starting point of the experiment (1.6 ± 0.081 ml KMnO_4_ g ^–1^ moist soil h^–1^) to the end of the second 20 days of incubation. However, in the third 20 days, a slight increase in this enzyme activity was detected compared to the second 20 days. The highest and lowest catalase activity during the experimental period was related to the NA treatment in the third 20 days (1.783 ml KMnO_4_ g ^–1^ moist soil h^–1^), and the SB treatment in the second 20 days (0.3 ml KMnO_4_ g^–1^ moist soil h^–1^), respectively (Figure 3C). Wu et al. (2016), reported that the higher the concentration of petroleum compounds is, the lower the catalase activity is. Instead, other investigation proved that the higher the concentration of petroleum compounds was, the higher the catalase activity was (Borowik et al., 2017).

Soil basal respiration, and microbial structure and population are acceptable criteria providing practical information on living microorganisms and demonstrating the long-term or short-term effects of contaminants on microorganisms (Schaefer et al., 2005). To provide a comprehensive definition of soil basal respiration, one should refer to all metabolic reactions obtained from the degradation of soil organic matter as a considerable indicator of microorganisms’ activity in the soil, especially in contaminated ones (Dos Santos et al., 2012). The results indicated that soil respiration was gradually increased from the beginning of the experiment (0.16 ± 0.008 mg CO_2_/kg soil/h) to the end of the 60th days of incubation in all treatments. Nonetheless, in the SB treatment, the upward trend continued as far as the end of the second 20 days, and then it was decreased. The maximum and the minimum amount of soil respiration was related to the BM treatment in the third 20 days (0.693 mg CO_2_/kg soil/h) and the SM treatment in the first 20 days (0.226 mg CO_2_/kg soil/h), respectively (Figure 3D). However, no significant difference in the respiration rate was observed between BM and SB treatments during the whole incubation period.

Toxic substances (petroleum-based hydrocarbons) can dramatically decrease the activities and the efficiency of degrader microorganisms, particularly when the organisms are inoculated into contaminated soil or water (Mariano et al., 2007). Therefore, an examination of the viability of the microorganisms provides us with pivotal information during biodegradation. Consistent with the initial soil bacterial population (1.4 ± 0.368 × 10^4^ CFU / g), the experiments showed that the largest and smallest bacterial population was recorded in the BM treatment in the second 20 days (4.3 × 10^6^ CFU / g) and the SM treatment in the third 20 days (2 × 10^3^ CFU / g), respectively (Figure 3E). During the incubation period, no significant difference was observed between BM and SB treatments. However, the SM treatment presented all the smaller microbial population than the NA treatment did. Margesin (2000) revealed that biostimulation had a significant adverse effect on the population of degrading microorganisms, being consistent with the results obtained in our study.

### Biodegradation of TPH in Crude Oil-Contaminated Microcosm

The older the soil organic contaminants are, the greater their pernicious lingering in the soil is, and the lower their degradability is (Hatzinger and Alexander, 1995). Under other conditions, in saline soils, the diversity and population of microorganisms remain low, and the solubility of petroleum compounds and oxygen reduces, which in turn can reduce the biodegradation of petroleum contaminants (Whitehouse, 1984). Moreover, considering the fact that this soil has been contaminated with crude oil for more than 60 years and has saline-sodic nature, removing the contamination from it is highly challenging and requires the adoption of various biological approaches. The results indicated that at the end of the 60th days of incubation, SB, BM, NA, and SM treatments reduced TPH by 38.2% ± 3.43, 34.4% ± 1.6, 18.2% ± 5.99, and 18% ± 3.11, respectively (Figure 3F). Notably, no significant difference was observed between SM and NA treatments at the end of the incubation period, and our results remained skeptical about the application of biostimulation alone as one of the bioremediation strategies. In this study, contrary to other reports by Kauppi et al. (2011), Wu et al. (2016), the biostimulation strategy had a negligible effect on the biodegradation of TPH even lower than NA had, which is due to the effect of surfactant (tween 80) on increasing the availability of contaminants (Cheng et al., 2008, 2017) and probably exposure of negligible soil microorganisms to excessive toxicity of these contaminants. Another point is that the preferential degradation of surfactants can also prevent contaminants’ biodegradation (Li and Chen, 2009). For instance, in an investigation reported by Bruheim et al. (1999), the use of the Corexit 9,527 reduced the biodegradation of alkanes in crude oil. Some reports demonstrate the opposite effect of Tween 80 on the biodegradation of contaminants (Pathak et al., 2009). Another assumption is that due to the large size of soil particles in this investigation (sandy-loam), contaminants are separated much easier from soil particles (Abdel-Moghny et al., 2012). However, the effect of the aforementioned and the surfactant usage might all the more increase the availability of contaminants, thereby exposing the small number of soil microorganisms to excessive toxicity. Interestingly, the results showed that in soils with a low population of native microorganisms, the use of biostimulation alone is not appropriate and capable microorganisms must be used for bioremediation process.

This study, as in some other studies, Garg et al. (2016), Roy et al. (2018), Varjani and Upasani (2019), Zeneli et al. (2019), shows that a combined strategy is the best technique to degrade hydrocarbon contaminants from the environment, especially in soils with old as well as excessive contamination. However, to achieve more significant biodegradation in old petroleum-contaminated soils, the use of potent microorganisms must be considered. Considering the limited reports on the performance of *B. altitudinis* in the bioremediation of hydrocarbon compounds (Yetti et al., 2016; Shahzad et al., 2020), this is the first report on the effectiveness of this strain in reducing TPH in highly contaminated aged soils with a high amount of salinity.

### Correlation and Analysis of Variance

The results of a correlation between soil biological indicators (enzymatic activities, soil basal respiration, and microbial population) and the amount of residual TPH in the microcosm environment showed a weak negative correlation for dehydrogenase activity (*r* = −0.34990, *P* = 0.0364), a moderate negative correlation for polyphenol oxidase activity (*r* = −0.43842, *P* = 0.0075), a moderate positive correlation for catalase activity (*r* = 0.48323, *P* = 0.0028), a strong negative correlation for soil respiration (*r* = −0.73915, *P* < 0.0001), and a moderate negative correlation for bacterial population (*r* = −0.65218, *P* < 0.0001). However, among the studied enzymes, dehydrogenase activity demonstrated the lowest correlation with the soil residual TPH content. Contrary to dehydrogenase and polyphenol oxidase activities with a weaker and negative correlation, catalase exhibited a stronger and positive correlation than the other two enzymes did. However, the results proved that the most reliable biological indicator in correlation with the soil residual TPH is the soil basal respiration.

The results of analysis of variance showed that the effects of treatment and time were individually significant for all microcosm indicators under investigation during the experiment period at (*P* ≤ 0.0001). The results of the interaction between treatments and times showed that catalase (*F* = 6.3, *P* = 0.0004) and soil basal respiration (*F* = 6.2, *P* = 0.0005) were significant at the 1% probability level (*P* ≤ 0.01), but the other biological indicators were not significant (dehydrogenase (*F* = 2.06, *P* = 0.096), polyphenol oxidase (*F* = 2.48, *P* = 0.052), microbial population (*F* = 2.08, *P* = 0.093), and biodegradation of TPH (*F* = 1.4, *P* = 0.255).

## Conclusion

In the present study, due to the long aging of soil contaminants, SB was the most effective and robust strategy to reduce TPH (38.2%) over a 60-day period. In heavily old aged petroleum-contaminated soils, considering the small number of native soil microorganisms as the main limiting factor for the bioremediation process, the biostimulation strategy alone was insufficient and not recommended. However, no significant difference in alleviating TPH was observed between SM and NA treatments at the end of the experiment. Regarding the vast difference in mitigating TPH between bacterial inoculated treatments and bio-stimulated treatment alone, it is concluded that the use of suitable microorganisms is necessary to reduce contamination in such soils. Therefore, the results indicated that the hydrophobic and biosurfactant-producing strain HRG-1 could play a pivotal role to remediate highly contaminated aged soils. The statistical analysis revealed that catalase had the highest correlation (positive) with the amount of soil residual TPH only in the studied enzymes. However, soil basal respiration indicated that it was more reliable than the other biological indicators were regarding its correlation with the amount of residual TPH in this soil with such a long history in terms of contamination.

## Data Availability Statement

The datasets presented in this study can be found in online repositories. The names of the repository/repositories and accession number(s) can be found in the article/Supplementary Material.

## Author Contributions

HG: conception and design of study, analysis and/or interpretation of data, writing and drafting the manuscript, and revising the manuscript critically for final version. AP: conception and design of study, analysis and/or interpretation of data, and revising the manuscript critically for final version. HA: interpretation of soil analysis. NY: interpretation of chemical analysis. All authors contributed to the article and approved the submitted version.

## Conflict of Interest

The authors declare that the research was conducted in the absence of any commercial or financial relationships that could be construed as a potential conflict of interest.
