# Supplementary material for: A Combinational Strategy Mitigated Old-Aged Petroleum Contaminants: Ineffectiveness of Biostimulation as a Bioremediation Technique
Source: Front Microbiol. 2021 Feb 25;12:642215. doi: 10.3389/fmicb.2021.642215 (PMC7947215; doi:10.3389/fmicb.2021.642215)
Supplement: Supplementary file 1 [file Table_3.DOCX]

Supplementary materials

Fig. S1. Biodegradation of different concentrations of crude oil in the modified 6SW-Vit medium after seven days of incubation at 40 °C temperature spectrophotometrically. Means with the same letters are not significantly different at (P ≤ 0.01). Error bars indicate the standard deviation (n = 3).


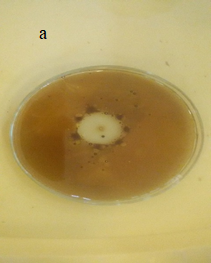


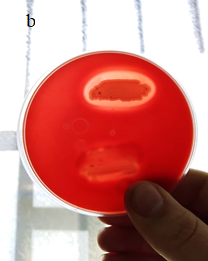


Fig. S2. Biosurfactant production assays by different methods a): oil displacement, b): lysis of red blood cells.
