# Supplementary figures and images for: A Combinational Strategy Mitigated Old-Aged Petroleum Contaminants: Ineffectiveness of Biostimulation as a Bioremediation Technique
Source: Front Microbiol. 2021 Feb 25;12:642215. doi: 10.3389/fmicb.2021.642215 (PMC7947215; doi:10.3389/fmicb.2021.642215)

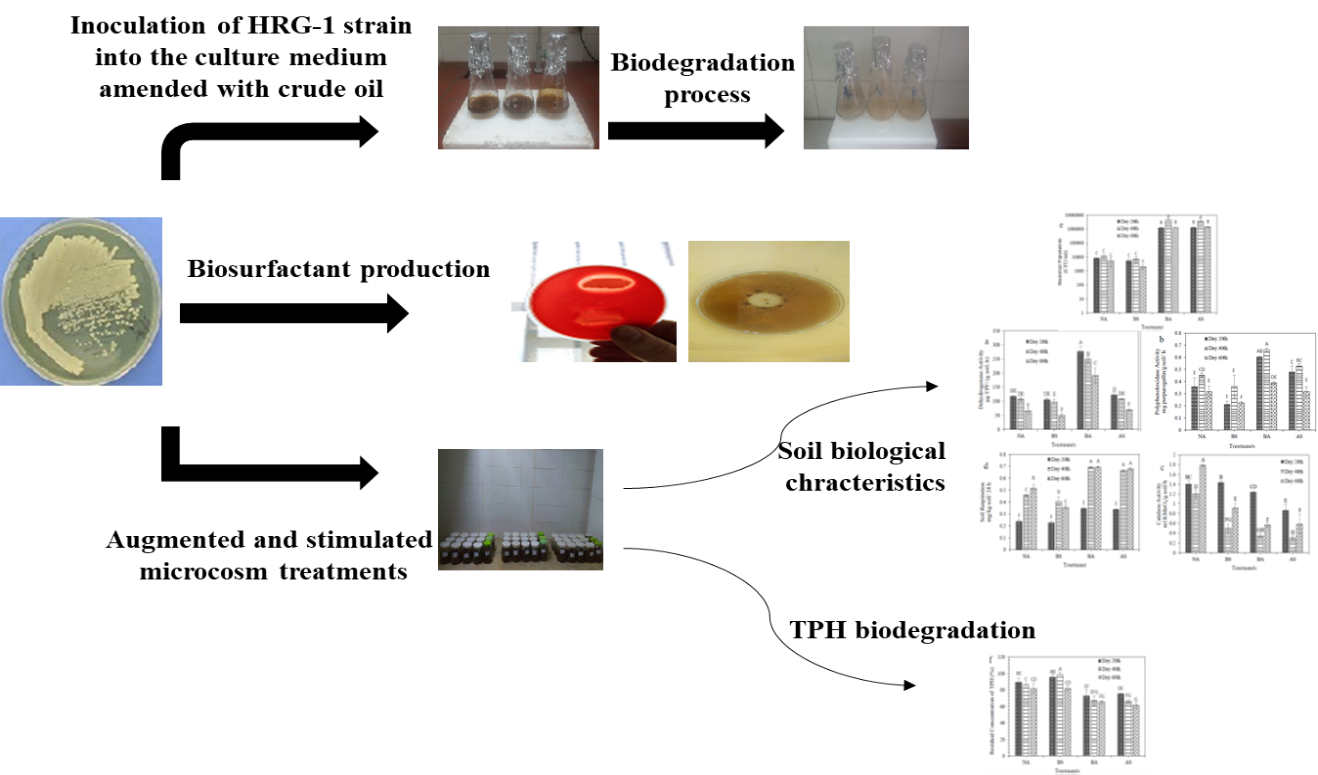

Supplement: Supplementary file 2 [file Table_4.DOCX]
